# VEGF as a Key Actor in Recurrent Respiratory Papillomatosis: A Narrative Review

**DOI:** 10.3390/cimb46070403

**Published:** 2024-07-01

**Authors:** Sandra Gazzini, Raffaele Cerullo, Davide Soloperto

**Affiliations:** 1 Division of Otolaryngology, Head and Neck Surgery Department, University Hospital of Verona, 37134 Verona, Italy; 2 Division of Otolaryngology, Hospital of Treviso, 31100 Treviso, Italy; 3 Department of Otorhinolaryngology, University Hospital of Modena, 41125 Modena, Italy

**Keywords:** recurrent respiratory papillomatosis, HPV, VEGF, angiogenesis, VEGFR2, VEGFR3, bevacizumab

## Abstract

Recurrent respiratory papillomatosis (RRP) is a benign disease of the upper aerodigestive tract caused by human papillomavirus (HPV) types 6 and 11. The clinical course is unpredictable and some patients, especially younger children, experience a high rate of recurrence with a significant impact on their quality of life. The molecular mechanisms of HPV infection in keratinocytes have been extensively studied throughout the years, with particular regard to its role in causing malignant tumors, like cervical cancer and head and neck carcinomas. A minor but not negligible amount of the literature has investigated the molecular landscape of RRP patients, and some papers have studied the role of angiogenesis (the growth of blood vessels from pre-existing vasculature) in this disease. A central role in this process is played by vascular endothelial growth factor (VEGF), which activates different signaling cascades on multiple levels. The increased knowledge has led to the introduction of the VEGF inhibitor bevacizumab in recent years as an adjuvant treatment in some patients, with good results. This review summarizes the current evidence about the role of VEGF in the pathophysiology of RRP, the molecular pathways activated by binding with its receptors, and the current and future roles of anti-angiogenic treatment.

## 1. Introduction

Recurrent respiratory papillomatosis (RRP) is the most common benign neoplasm of the larynx, caused by chronic infection with human papillomaviruses (HPVs) [1]. While its incidence has classically been estimated as 1–4 cases per 100,000 persons, more recent studies have shown a decrease in incidence after the systematic introduction of the HPV vaccine, with values of 0.5–0.7 per 100,000 [2]. HPVs are classified as high risk (e.g., HPV 16, 18, and 31) or low risk (e.g., HPV 6 and 11), based on their capacity to cause cancer [3]. Infection with a low-risk virus in humans can result in the formation of benign lesions, such as papillomas or skin warts, while infection with a high-risk virus may lead to the development of carcinomas of the cervix or oropharynx.

RRP is mainly caused by infection with low-risk HPV 6 and HPV 11, and some risk factors are believed to be involved in the infectious process, such as maternal anogenital warts and low socioeconomic status (in juvenile form), and a high number of sexual partners and oral sex (in adult form) [4]. Moreover, the course of the disease can vary among individuals, ranging from an indolent course with even spontaneous remission (less frequently) to an aggressive and recalcitrant disease with many recurrences after treatment [5]. RRP also shows a low rate (1–2%) of malignant transformation to squamous cell carcinoma, but the underlying mechanisms are poorly understood [6]. The treatment of this disease still mostly consists of repeated surgical excision of papillomatous lesions, which has recently been joined by some adjuvant treatments, although many are still off-label [5]. The HPV vaccine that targets HPV6 and HPV11 (Gardasil9) has sometimes been used as an adjuvant therapy in recent years, showing encouraging results in reducing the number of recurrences [7]. Two in-human phase I/II clinical trials on the adjuvant use of HPV vaccines are ongoing: NCT04398433 and NCT04724980, which investigate the benefits of INO-3107 (in both adults and adolescents) and PRGN-2012 (in adults) vaccines, respectively [8,9]. INO-3107 consists of DNA plasmids combined with an electroporation device, and PRGN-2012 consists of an optimized antigen with a gorilla adenovector. They both enhance specific T-cell immunity against HPV-6 and HPV-11, and interim results are showing promising clinical benefits.

Many studies on the pathophysiology of RRP have focused on the role of HPV and the interaction of viral proteins with the immune system [10]. The most investigated viral proteins involved in the disease process and in the viral cell cycle are E6 and E7, which bind the oncosuppressors p53 and retinoblastoma (Rb) as main targets, respectively. E6 sends p53 to degradation, whereas E7 targets Rb, thus altering the DNA repair pathways [11]. This can contribute to the development of cancer, especially when infected with high-risk genotypes, while E6 and E7 of low-risk genotypes do not cause genomic instability [12,13]. These proteins are also involved in the development of an imbalanced immune response by interacting with cytokines, chemokines and interferons (IFNs) [14]. This ultimately leads to reduced cytotoxic T-cell activity against HPV and promotes viral immune evasion and persistent infection.

Recent studies have focused on the molecular process of angiogenesis, an important step in the development of tumors, which consists of the ability of cells to recruit a blood supply by forming new vessels from pre-existing vasculature [15]. This is of paramount importance for inflammation and wound healing, but in the case of neoplasms, it allows the tumor to obtain the necessary nutrients and oxygen for sustaining its growth and expansion [15,16]. 

Angiogenesis is an early event in the tumorigenesis process and results from the decreased expression of angiogenesis inhibition factors and/or an increase of inducers [15]. 

A key role in this process is played by vascular endothelial growth factor (VEGF), a signal protein that stimulates endothelial cell growth and increases vascular permeability [16,17]. VEGF signaling is mediated by interactions with specific tyrosine kinase receptors, which can activate different pathways, like the Raf-MEK-ERK signaling cascade and the phosphatidylinositol-4,5-bisphosphate 3-kinase (PI3K)/protein kinase B (Akt) pathway [18]. These pathways enhance cell migration, vascular permeability, and cell survival.

The increased knowledge about the molecular mechanisms involved in the pathophysiology has led to the development of targeted therapy against angiogenesis, with a direct or indirect inhibition of this process [19]. The first anti-angiogenic drug approved by the FDA for the clinical treatment of tumors was bevacizumab (Avastin^®^) as second-line therapy for colorectal cancer in 2004 [20]. Thereafter, the indications for these drugs in clinical practice have rapidly expanded, including mainly malignant tumors [19]. The appearance of bevacizumab on the RRP treatment scene as an adjuvant treatment in recent years has given a boost to a growing literature about this topic, showing promising results that could lead to the approval of this drug for clinical practice in the future [21,22]. 

The aim of this paper is to review the current literature about the molecular mechanisms and the genomics and proteomics associated with VEGF expression in RRP cells.

## 2. Biology of VEGF Signaling

VEGFs comprise a family of proteins which includes four isoforms in mammals (VEGF-A,B,C,D) and placental growth factor (PIGF) [23]. The functional protein binds to the specific VEGF receptor (VEGFR), a family of tyrosine kinase receptors expressed on the surface of endothelial cells which generate signal transduction [24]. Given the dominant role that VEGF-A plays in the angiogenesis process and the large number of papers on this isoform in the literature, we will mainly focus on this protein.

VEGF-A is the most potent agent for vascular permeabilization. It interacts with two receptors, VEGF-A receptor-1 (VEGFR-l) and VEGF-A receptor-2 (VEGFR-2), which are highly expressed on vascular endothelium. Through this interaction, VEGF-A increases vascular permeability to plasma within seconds to minutes, with subsequent extravasation of plasma proteins and stromal changes, leading hours to weeks later to endothelial cell changes and migration to create new vessels [25]. 

The role of VEGFR-1 in angiogenesis is still not clear, since a unique expression pattern has not been observed after its stimulation. It has weak ligand-dependent tyrosine autophosphorylation but seems sometimes to prevent VEGF binding to VEGFR-2, although studies have demonstrated that the two receptors appear to cooperate in inducing gene expression in endothelial cells [26]. 

However, other studies have revealed that VEGFR-1 may play a role in the tissue-specific release of growth factors from sinusoidal endothelial cells in the liver, with a protective role for hepatocytes in hepatic insult [27]. In addition, activation of VEGFR-1 in monocytes and macrophages has been reported to mediate the migration of these cells [28]. 

VEGFR-2 is a tyrosine kinase receptor consisting of an extracellular ligand-binding domain, a transmembrane domain, and an intracellular kinase domain that is activated upon ligand binding; it binds VEGF with high affinity and presents two tyrosine residues that promote and regulate endothelial cell mitosis and vascular permeability, respectively. [26,29]. 

This receptor has one or more domains that allow interactions with signal-transmitting proteins at the intracellular level, e.g., the SH2 domain that allows binding to phosphotyrosine residues [30]. 

In fact, binding to VEGF results in conformational changes in VEGFR-2: the intracellular N-lobe binds ATP and consequently allows intrinsic receptor kinase activity and phosphorylation of tyrosine residues in the C-lobe. This event creates binding sites for cytoplasmic proteins that transduce the signal intracellularly. These signaling pathways include proliferation via phospholipase C-γ (PLC-γ) and extracellular signal-related kinase 1/2 (ERK1/2), focal adhesion kinase (FAK)-mediated cell migration, and cell survival through phosphatidylinositol-4,5-bisphosphate 3-kinase (PI3K)/protein kinase B (Akt) [31].

When phosphorylated by VEGRF-2 binding, PLC-γ is activated and catalyzes the hydrolysis of phosphatidylinositol 4,5-bisphosphate (PIP2) to inositol 1,4,5-trisphosphate (IP3) and diacylglycerol (DAG). These two molecules have a role in releasing Ca^2+^, which in turn leads to the production of nitric oxide and prostacyclin (PGI2). Another action of DAG is the activation of protein kinase C (PKC), a serine-threonine kinase that stimulates endothelial cells proliferation via the Raf-MEK-ERK pathway [30,32]. 

On the other hand, FAK activation stimulates the recruitment of actin-anchoring proteins to the focal adhesion plaque, a phenomenon underlying cell migration.

Another important enzyme is PI3K, which presents a p85 regulatory subunit that is phosphorylated by VEGFR-2; this increases PI3K activity, which has a role in cell migration, proliferation, and survival. In fact, activation of PI3K determines the increase in intracellular levels of phosphatidylinositol-3,4,5-trisphosphate (PIP3), with consequent phosphorylation and activation of Akt/PKB. Akt/PKB in turn phosphorylates proapoptotic proteins, such as BAD, FKHR1, and caspase-9, with an inhibitory effect [30,33].

Moreover, PI3K induces endothelial cell migration, probably through the action of the Rho family of small GTPases. However, the exact signal transduction is currently unknown [30].

Important products of VEGFR-2 stimulation are NO (nitric oxide) and eNO (endothelial nitric oxide), molecules implicated in endothelial cell proliferation, migration, tube formation, increased vascular permeability, and angiogenesis [34]. eNO is activated directly by Akt/PKB, but also via PKC and as a consequence of Ca^2+^ release from intracellular reserves [30]. 

**Figure 1:**
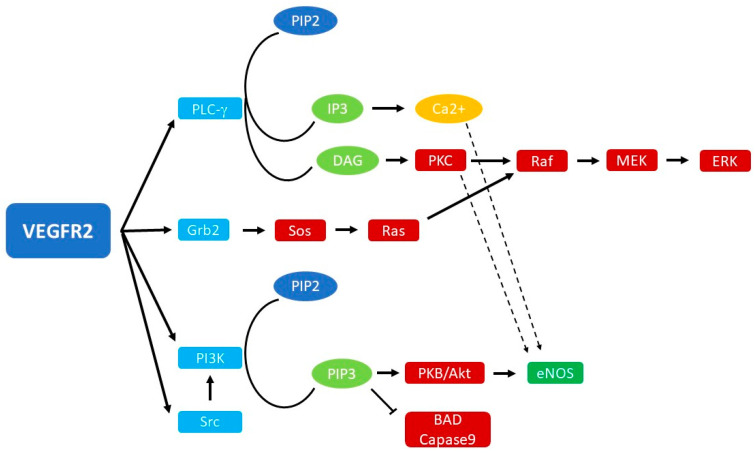
Summary illustration of signaling pathways induced by VEGFR2 activation.

In fact, it must be remembered that these signal transduction pathways do not work as watertight compartments but intersect with each other. An overview of the signal transduction pathways activated by VEGFR-2 is shown in Figure 1.

**Figure 2:**
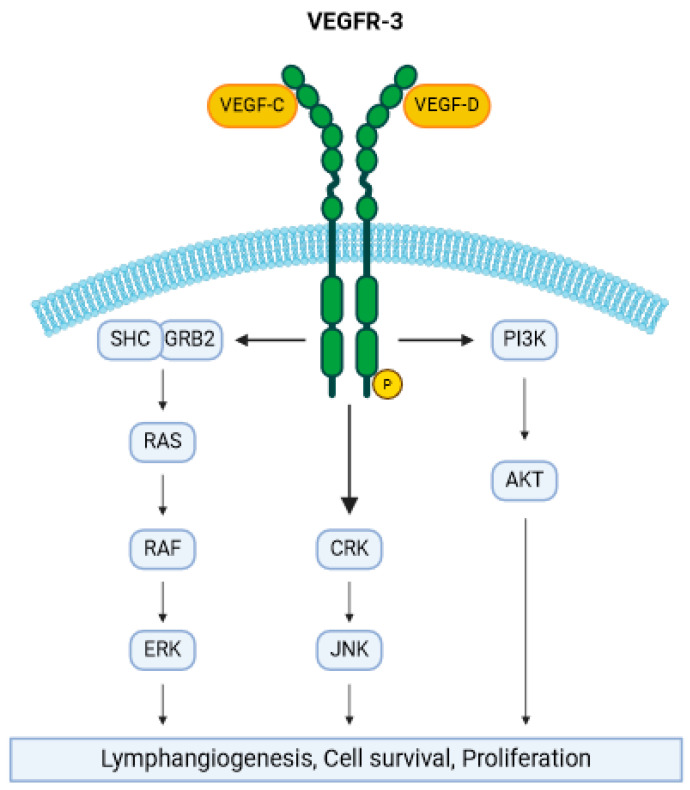
Schematic illustration of the molecular pathways induced by VEGFR-3 activation. The tyrosine kinase receptor acts as a homodimer and, after autophosphorylation, induces many distinct molecular pathways, such as the RAS/RAF/ERK pathway, JNK activation, and the PI3K/AKT pathway. This promotes cell survival, lymphangiogenesis, and proliferation. The figure was created with BioRender.com.

A less studied but no less important receptor of VEGF is VEGFR-3, a tyrosine kinase that has a higher affinity for VEGF-C and VEGF-D [35]. Its expression in human adults is limited to lymphatic endothelial cells and regulates transient lymphatic angiogenesis in inflammation. After binding with its ligand, VEGFR-3 acts as a homodimer and activates SRC homology domain-containing (SHC) and growth factor receptor-bound protein 2 (GRB2), which in conjunction with PI3K activate various pathways such as the PI3K/MAPK-associated family members AKT, ERK1/2, and JNK (c-Jun N-terminal kinase) [36]. All these pathways stimulate endothelial and lymphatic cell proliferation and survival, as summarized in Figure 2. Some authors have also postulated a negative modulation role for VEGFR-3 in VEGFR-2 signaling of endothelial cells to maintain vascular integrity [37]. Hereditary functional mutations of VEGFR-3 have been associated with hereditary lymphedema (Milroy disease) and, interestingly, some patients with this rare disease show skin papillomatosis [38,39]. In some tumoral cells, VEGFR-3 upregulation has been shown to enhance tumoral lymphangiogenesis and metastatic spread to regional lymph nodes [40]. On the other hand, inhibition of VEGFR-3 signaling may suppress lymphangiogenesis and lymph node metastasis [41]. 

In summary, the increased understanding of the VEGF-VEGFRs pathway in recent years has provided a more comprehensive overview of the complex mechanisms underlying both physiological and pathological angiogenesis.

## 3. Role of VEGF in HPV-Mediated Diseases and RRP

Histologically, papillomas in RRP are composed of vascular projections covered by stratified squamous epithelium. Different studies have shown the expression of VEGF-A mRNA and its receptors in the squamous epithelium and endothelial cells of papillomas and increased levels of serum VEGF-A in RRP patients [16,25]. In addition, Verma et al. [16] demonstrated a linear correlation between the expression of VEGF and disease extension, with higher systemic and local expression of VEGF in more aggressive RRP cases.

Moreover, as shown in the recent work by Lam et al., [42] the expression of VEGFR3 is significantly increased in papilloma tissue compared to normal adjacent tissue (while VEGFR2 did not show this overexpression), suggesting that VEGF signaling in RRP could be mediated by this specific receptor.

These findings may suggest that angiogenesis plays a role in the formation of papillomas, which is confirmed by other studies that focused specifically on the interaction between HPV proteins and VEGF signaling [43]. 

As already described, E6 and E7 are the most studied HPV proteins, and they interact both directly and indirectly with VEGF signaling, while a minor role is played by the weak malignant transforming protein E5. E6 sends p53 to degradation through activation of the cellular ubiquitin ligase E6AP, while E7 activity leads to activation of the elongation factor 2 (E2F) and increased expression of the cellular p16, and ultimately enhancing cell proliferation [44,45]. 

Tumor suppressor p53 has the role of inhibiting cell growth and malignant transformation, participating in a checkpoint mechanism that can arrest the cell cycle in the G1 phase after genotoxic insult, permitting the repair of damaged DNA [46]. 

It is well established in the literature that p53 also has a role in repressing VEGF expression, through inhibition of transcriptional factors such as SP1 or forming a transcriptional repressor complex with elongation factor 2 (E2F) transcription factor, but there is evidence that the relationship between p53 and VEGF is not so linear. In fact, p53 is also involved in the expression of VEGF during the initial phases of hypoxia [47,48]. It can complex with hypoxia-inducible factor-1a (HIF-1a, a major regulator of the hypoxic response) and bind a specific promoter region of VEGF, inducing its transcription [47,48,49].

Ghahremani et al. [48] demonstrated that p53 can positively regulate VEGF expression through the bond with HIF-1a during initial acute hypoxia, but prolonged hypoxic conditions determine VEGF repression. This happens through the p21/E2F/Rb pathway. E2F protein is expressed at low levels in the initial phases and increases during the final stages of hypoxia, binding p53 and subsequently decreasing VEGF expression. In addition, after cytotoxic insult, the level of p53 increases, which brings about an increase in p21 levels. p21 stops the phosphorylation of the Rb protein (when dephosphorylated, the Rb protein acts as an inhibitor of progression in the cell cycle) and decreases the level of free E2F. However, in the case of prolonged hypoxia, p21 levels remain constant and phosphorylation of Rb is unchanged, inactivating the protein; the levels of free E2F increase, which complex with p53, acting as an indirect repressor of VEGF expression [48,49].

Apart from the p53-dependent mechanism, some authors have shown that E6 can stimulate VEGF expression in a direct manner. This happens by binding four SP1 sites between −94 and −50 bp in the VEGF promoter [50].

In a parallel fashion, E7 increases VEGF expression through telomerase reverse transcriptase (hTERT) and telomerase activity, with two independent mechanisms [51,52]. In particular, overexpression of hTERT upregulated VEGF expression in HPV-18-positive HeLa cells, and knockdown of hTERT expression downregulated VEGF expression. However, the mechanism of hTERT activation in HPV-infected cells has yet to be fully elucidated, since some authors have postulated a role of E6 in its activation in an SP1-dependent way [53]. 

**Figure 3:**
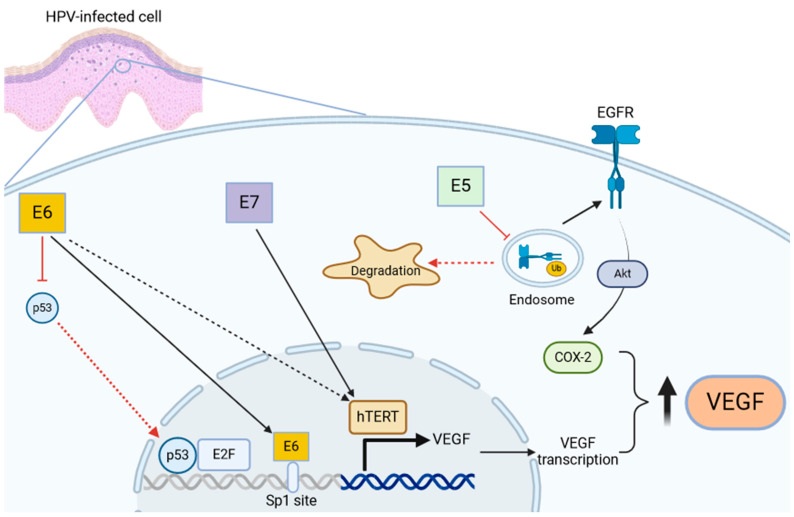
Schematic illustration of the hypothesized role of E5, E6, and E7 proteins in increasing levels of VEGF in HPV-infected cells. E5 induces EGFR expression by reducing its ubiquitination and degradation, thereby activating COX-2, which induces VEGF production. E6 blocks the interaction of p53 with E2F, which reduces VEGF transcription; furthermore, E6 directly induces VEGF transcription in a p53-independent way by binding specific sites on the VEGF promoter. Finally, E7 activates hTERT, which induces VEGF transcription. The figure was created with BioRender.com.

A minor role in VEGF expression during HPV infection could be played by E5, an oncoprotein that activates the epidermal growth factor receptor (EGFR), with a poorly understood mechanism, which might involve interaction with the vacuolar H^+^-ATPase, reducing acidification of the endosomal compartment and preventing EGFR degradation according to some authors [54]. Moreover, E5 reduces ubiquitination of EGFR and then increases its expression [55]. This causes the phosphorylation of phosphatidylinositol 3-kinase (PI3K) and Akt, which increases VEGF expression by activating the transcription of COX-2 [56,57]. An overview of the described mechanisms of VEGF activation in HPV-infected cells is illustrated in Figure 3.

These pathways only partly explain the delicate molecular equilibrium that regulates VEGF expression. Certainly, other factors are also involved, through mechanisms that are currently unknown. What is certain is that HPV proteins E6 and E7 (and, to a lesser degree, E5) alter this equilibrium in a pro-angiogenic manner, which is confirmed by the discovery of high levels of VEGF in papillomatous epithelium and underlying endothelial cells in RRP patients. Moreover, the increased expression of VEGF, along with angiogenic chemokines CXCL1 and CXCL8, in RRP cells could be a marker of disease severity [58].

## 4. Anti-Angiogenic Molecules in The Treatment of RRP: Present and Future

Based on the observation of the presence of VEGF mRNA and its receptors in the squamous epithelium of papillomas and endothelial cells, in 2009, Nagel et al. [21] empirically administered bevacizumab, an anti-genetic agent, to a young patient with RRP, with excellent results in terms of disease control and improved quality of life.

The patient, a 32-year-old male, had been suffering from recurrent laryngeal papillomatosis since the age of 2 years and underwent numerous surgical ablations and tracheal dilatation surgeries due to frequent recurrences and subsequent tracheal stenosis. Despite numerous laser treatments and mechanical endobronchial ablation of the lesions, placement of tracheal stents, and an attempt at therapy in the form of local application of 30 million IU of Roferon and local instillation of cidofovir, recurrences were appearing about 8 weeks after each treatment. The authors then decided to put the patient on empiric therapy with bevacizumab, and after only 8 cycles of treatment, they observed good control of the disease. The patient remained in prolonged remission, and no further endobronchial excisions were necessary.

Bevacizumab is a recombinant humanized monoclonal antibody that binds VEGF-A and inhibits its activity by preventing binding to the receptor VEGF-R and consequently its activation [59,60]. This causes a decrease in vessel diameter, density, and permeability, leading to regression of existing microvessels, normalization of the mature vasculature, and inhibition of neovascularization. This brings about improvement of the metabolic microenvironment [61,62,63]. 

To date, bevacizumab has been approved, in combination with chemotherapy drugs, for the treatment of metastatic colorectal cancer, breast cancer, non-squamous cell lung cancer, metastatic or advanced renal cell cancer, ovarian cancer, and advanced glioblastomas [62].

Recent studies have hypothesized a role for this therapy not only in oncology but also in treating all those pathologies characterized by altered angiogenesis, for example, in retinal capillary hemangiomas, hereditary hemorrhagic telangiectasia or intracranial arteriovenous malformations, with a good response in terms of control or regression of the pathology [61,62,63,64,65,66].

Since the first study investigating its use in RRP, bevacizumab has been considered a promising therapy in RRP as well, both as monotherapy and as an adjuvant therapy to surgery, especially in cases of severe disease with invalidating symptoms and frequent recurrences [67]. 

Many authors describe an impressive response to therapy, in terms of a reduction in surgical interventions and lengthening of intersurgical intervals. In most cases, only a partial response was evident, with a reduction in the exophytic component of papillomas without their complete elimination. However, this led to an important reduction in airway obstruction, with marked improvement in quality of life, a reduced number of surgical procedures required per year, and improvement in voice quality [59,67,68,69]. 

Mohr et al. [70] described the resorption of perivascular edema in the absence of apoptosis of papillomatous tissue after bevacizumab administration. This confirms the role of the drug in acting on the vasculature without inducing cell apoptosis.

Bevacizumab can be administered either systemically intravenously or intralesionally. The former is necessary in cases of non-accessible bronchial lesions, involvement of lung parenchyma, or risk of voice mutilation due to surgery, while the latter is indicated in the case of laryngeal lesions [60,71]. 

When therapy is discontinued, progression of the disease has been seen within a few months. However, resumption of treatment again leads to a good response. To date, surgical treatment cannot always be completely avoided in patients with severe RRP, but the interval between surgeries appears lengthened [67,70]. 

Regarding adverse effects, intralesional administration of bevacizumab seems to be devoid of complications [71]. With regard to systemic administration, even fatal adverse effects, such as bleeding, intestinal perforation, proteinuria, and neutropenia, are described in the literature when bevacizumab is used in cancer patients in combination with chemotherapy drugs. However, these adverse effects appear to depend on some specific factors, such as the histologic type of tumor, the associated chemotherapy drug and the dose of bevacizumab administered [72,73,74]. 

In contrast, adverse effects in the case of systemic treatment with bevacizumab in RRP are described as mild and self-limiting. This could indicate the safety of the treatment, but long-term follow-up and a shared therapeutic protocol for RRP are still lacking [22]. 

In addition, some concerns have been raised about the costs of using Avastin in clinical practice. An option to reduce the economic impact of this therapy could be the use of biosimilars (like SB8 and ABP 215) [75,76], which some authors have shown can significantly reduce treatment costs and improve cost-effectiveness. However, the specific cost-effectiveness of biosimilars in RRP patients has not been investigated yet and could be an additional aspect to be explored in future research [77]. 

In addition to bevacizumab, the use of other anti-angiogenic drugs is described in the literature.

Although there are many molecular mechanisms involved in angiogenesis, research has mainly focused on the VEGF/VEGFR signaling pathway, which is most involved in this process [19]. 

Anti-angiogenic drugs approved by the FDA for clinical treatments include monoclonal antibodies, which are characterized by high binding specificity (in addition to bevacizumab, this class also includes ramucirumab, which binds the extracellular domain of VEGFR-2, preventing its binding to VEGF, and ranibizumab, which binds VEGF), anti-angiogenic recombinant fusion proteins (such as aflibercept, which binds VEGF by preventing its binding to the receptor), and small-molecule kinase inhibitors. The latter are competitive non-covalent ATP inhibitors that inactivate VEGFR; included in this category are imatinib, sorafenib, sunitinib, pazopanib, vandetanib, axitinib, regorafenib, and many others [19,78]. 

These drugs are already in use, as monotherapy or in combination with chemotherapeutics, for advanced gastric metastatic carcinoma, colorectal carcinoma, kidney, liver, thyroid, pancreatic cancers, gastrointestinal solid tumors, and non-small cell lung cancer. In addition to neoplastic diseases, they are also used for angiogenic age-related macular degeneration, diabetic retinopathy, and other oculopathies [19,78].

Regarding RRP, the only drug used so far in the literature is bevacizumab, which has shown promising results. However, the application of other anti-angiogenic drugs that act on the VEGF/VEGRF pathway could also be studied in RRP in the future.

Furthermore, given the importance that is being attributed in recent years to VEGFR-3 in regulating the angiogenesis process [79], it can be hypothesized that this could become a target of anti-angiogenic therapy in the future, alone or in combination with selective inhibitors of the HPV-infected cell cycle.

## 5. Conclusions

VEGF seems to be an important actor in the pathophysiology of many viral diseases, including RRP, in which angiogenesis plays a crucial role, especially in more severe cases. Overexpression of VEGF is surely part of the genetic dysregulation in the papilloma microenvironment, with patterns unique to every patient. The mechanisms of this upregulation are several, and the viral oncoproteins E5, E6, and E7 are directly involved in many of them.

After the successful use of bevacizumab as an adjuvant therapy for RRP in reducing the number of recurrences, few papers have studied the mechanisms underlying the pro-angiogenic shift in RRP. However, the literature on this specific topic is still limited and future research could investigate more specifically the genomics and proteomics in every cellular lineage in the papilloma microenvironment, since not every patient shows increased expression of proteins involved in VEGF signaling, suggesting that other pathways could be involved. The final aim is the creation of a tailored combination therapy for each patient, which can block multiple pathological pathways at the same time. The reduced tumor escape could decrease the number of recurrences and surgical procedures performed, promoting a better quality of life for these patients.
